# An outbreak of bovine babesiosis in February, 2019, triggered by above average winter temperatures in southern England and co-infection with *Babesia divergens* and *Anaplasma phagocytophilum*

**DOI:** 10.1186/s13071-020-04174-3

**Published:** 2020-06-12

**Authors:** Nicholas Johnson, L. Paul Phipps, Harriet McFadzean, Alex M. Barlow

**Affiliations:** 1 Virology Department, Animal and Plant Health Agency (Weybridge), Woodham Lane, Addlestone, KT15 3NB Surrey UK; 2 Faculty of Health and Medical Science, University of Surrey, Guildford, GU2 7XH Surrey UK; 3 Veterinary Investigation Centre Starcross, Animal and Plant Health Agency, Staplake Mount, Starcross, Exeter, EX6 8PE Devon UK; 4 Wildlife Network for Disease Surveillance, School of Veterinary Science, University of Bristol, Langford, Somerset, BS40 5DU UK

**Keywords:** Babesiosis, *Babesia divergens*, *Anaplasma phagocytophilum*

## Abstract

**Background:**

Bovine babesiosis, commonly known as redwater fever, is a sporadic tick-borne disease in the United Kingdom. Outbreaks occur during the spring, summer and autumn months when ticks are active. This study reports the findings of an investigation of an outbreak of bovine babesiosis during the winter month of February, 2019.

**Methods:**

DNA from blood, organ and tick samples taken from affected cattle were tested for the presence of piroplasm and *Anaplasma phagocytophilum* DNA using PCRs directed to the *18S* rRNA gene and *msp2* gene respectively. The species of piroplasm was confirmed by sequencing.

**Results:**

*Babesia divergens* DNA was detected in the blood of five cattle displaying clinical signs of babesiosis within a herd of twenty. This parasite was also detected in three of ten ticks removed from one of the affected cattle. In addition, *A. phagocytophilum* was detected in three cattle tested and two of ten of the ticks.

**Conclusions:**

An outbreak of bovine babesiosis during February is unusual as the tick vector, *Ixodes ricinus*, does not generally become active until temperatures rise later in the year. February of 2019 was unusual as average temperatures during the first week of the month reached over 10 °C, well above historical averages that are typically below 5 °C, and a temperature at which ticks can become active. This unusual weather event is likely to have triggered tick questing, that combined with a co-infection with two tick-borne pathogens caused the severe outbreak of disease.
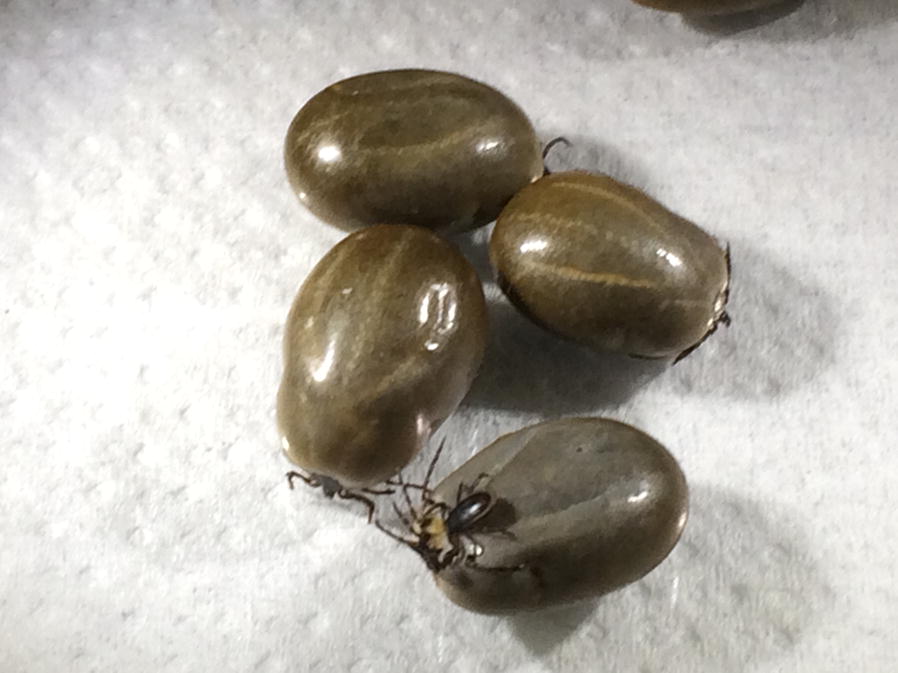

## Background

Bovine babesiosis is a tick-borne disease of cattle that causes significant morbidity and mortality in the United Kingdom (UK) and Ireland [1]. The disease occurs sporadically with clinical signs ranging from fever and anorexia in mild cases, to a fatal condition with haemolytic anaemia, dehydration, diarrhoea and weakness [2]. The destruction of red blood cells by high parasite loads leads to release of haemoglobin that is passed in urine giving the disease its common name of redwater fever. A number of species within the genus *Babesia* cause bovine babesiosis with two, *B. divergens* [3] and *B. major* [4] being reported in the UK. The tick vector of *B. divergens* is the common sheep tick or pasture tick, *Ixodes ricinus*, a vector found across Europe [5].

Reports of bovine babesiosis in the UK have been documented infrequently throughout the past one hundred years. One outbreak of redwater on a Sussex farm in 1969 resulted in the death of three dry Friesian cows in early July and August from a herd of 56 cows [6]. *Babesia divergens* was confirmed by blood smear from the affected animals. A more recent case occurred in September, 2016, affecting Holstein cows in Cumbria [7]. However, most outbreaks of disease go unrecorded and the factors that lead to its occurrence are poorly studied. Anecdotal evidence suggests that cattle movement is a key driver leading to outbreaks of bovine babesiosis. Seasonal incidence data based on reports from the county of Devon in the south-west of England showed that cases were reported during the spring months (March onwards), peaking in May and declining until November [8]. This reflects the seasonal activity of the tick vector in the UK [9].

This case study reports an outbreak of bovine babesiosis in a herd of cattle in Dorset in February, 2019. Blood samples, organ samples removed at necropsy and engorged ticks removed from a carcass were submitted to the Animal and Plant Health Agency (APHA) Weybridge laboratory for molecular testing for *Babesia* infection. The outbreak may have resulted from the unusually elevated temperatures experienced in southern England during the month of February that triggered questing activity in the resident tick population and the movement of *Babesia*-naive cattle onto tick-infested fields.

## Methods

A range of samples including EDTA treated blood, organ samples (liver and kidney) and ten engorged female ticks were submitted to APHA Weybridge for testing as listed in the Table. In addition, a number of males associated with the females had been included. The ticks were identified morphologically as *Ixodes ricinus*. Total DNA was extracted from 200 µl of blood, 0.1 g solid tissue or four legs removed from each engorged female tick using the QIAamp RNeasy kit (Qiagen, Manchester, UK) following the manufacturers methods. DNA was eluted in a volume of 200 µl of elution buffer supplied with the kit.

DNA samples (5 µl) were tested for the presence of *Babesia* species using two pan-piroplasm polymerase chain reaction (PCR) tests as previously reported [10]. These assays target the *18S* rRNA and cytochrome *c* oxidase subunit 1 (*cox*1) genes and produce a 350-bp and 150-bp amplicon, respectively. These primer pairs amplify a wide range of *Babesia* spp. and *Theileria* spp. and is effective when the identity of the infecting piroplasm is not known. DNA containing *B. caballi* genome was used as a positive control. Amplicons were separated on a 1.5% agarose gel impregnated with Sybr® Safe DNA gel stain (Thermo Fisher Scientific-UK, Horsham, UK) and visualised under UV illumination. Band sizes were determined by comparison with Gel pilot 50 bp DNA ladder (Qiagen). Sequences of the sample amplicons were produced using flanking primers and edited using Laser gene version 12.1 (DNASTAR, Madison, USA). The *Babesia* species was identified using a BLAST search (NCBI).

DNA extraction was performed on EDTA treated blood samples and from tick samples. These were also tested for the presence of *Anaplasma phagocytophilum* (by *msp2* gene segment targeting) using a real-time PCR. DNA extracted from a tick infected with *A. phagocytophilum* from a prior study was used as a positive control. The method of testing is based on a previously described protocol by Courtney et al. [11].

## Results and discussion

**Fig. 1:**
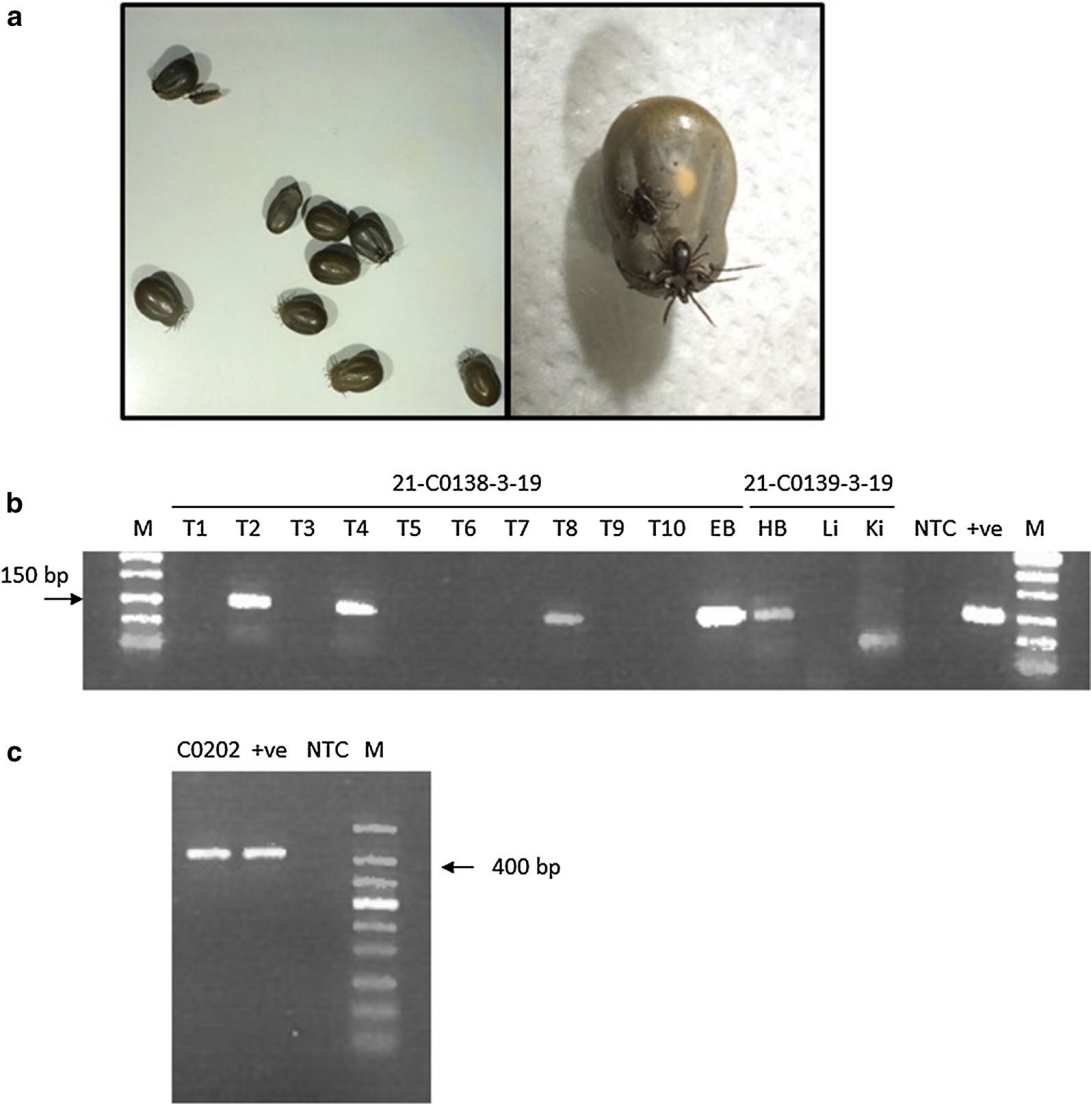
Detection of piroplasm infection within cattle blood samples and engorged *Ixodes ricinus* ticks removed from *Babesia-*infected cattle, 2019. **a** Photographs of engorged female *I. ricinus* ticks (left panel) and one engorged female with two male *I. ricinus* ticks attached (right panel). **b** Pan-piroplasm PCR (BbMit3/2) on tick (T1-T10) and blood/organ samples removed from a suspect cow. **c** Pan-piroplasm PCR (PIROA/B) on sample C0202-03-19 (see Table 1). *Abbreviations*: C0202, cow sample C0202; EB, EDTA-treated blood; HB, blood removed from heart; Li, blood removed from liver; Ki, blood removed from kidney; NTC, no-template control; +ve, *Babesia* positive control; M, DNA markers

Early in February of 2019, a herd of 20 beef cows with calves at foot were moved onto an area of extensive conservation grazing pasture where they are believed to have first encountered ticks. Towards the end of the month, six cows developed clinical signs including depression, recumbency, anaemia and jaundice. Large numbers of ticks were reported on affected animals. One animal, which also presented with haematuria characteristic of babesiosis, was killed on welfare grounds and the carcass submitted for necropsy. Samples of blood, organ tissue and ticks (see Table 1 and Fig. 1a) were removed and submitted for molecular confirmation of babesiosis. Blood samples from a further three symptomatic cattle were also submitted. These animals later died. Blood samples from the necropsied cow and three other cows from the herd were positive by pan-piroplasm PCR (Fig. 1b). A further sample of blood removed from the heart of the necropsied cow was also weakly positive. However, samples of liver and kidney were negative when tested. DNA extracted from leg samples removed from each of the ten female ticks produced positive results in three samples (Table 1 and Fig. 1b). *18S* rDNA sequences derived from both the blood and tick amplicons (Table 1 and Fig. 1c) were identical to each other and showed 100% identity with a *B. divergens* sequence on GenBank (accession number MG344781).

**Table 1:** Results of pan-piroplasm and *A. phagocytophilum* PCR of cow and tick samples

Sample code	Sample type	Pan-piroplasm PCR	Sequencing	Sequence analysis	*A. phagocytophilum* PCR
C0124 03 19	EDTA blood	+	nd	nd	nd
C0138 03 19	EDTA blood	+	+	Sequence 100% identity to *B. divergens* (GenBank: MG344781)	+
C0138 03 19	Engorged ticks (×10)	+ (tick #2, #4, #8)	+++	All three 100% identity with C0138-03-1	+ (tick #1, #5)
C0139 03 19	Heart blood	+			nd
	Liver	–			
	Kidney	–			
C0202 03 19	EDTA blood	+	nd	nd	+
C0204 03 19	EDTA blood	+	nd	nd	+

*Abbreviation*: nd, not done

Given the high morbidity rate and the normal appearance of urine in multiple affected cows, testing for additional contributing factors was carried out. *Anaplasma phagocytophilum* DNA was detected by real-time PCR on EDTA blood samples in the necropsied cow, along with two symptomatic cows. In addition, two of the ticks submitted for testing were also positive for *A. phagocytophilum* although dual infection with both pathogens was not observed in the ticks. This is perhaps not surprising given the small sample size used in this study. However, even large surveys of ticks do not detect co-infection with *B. divergens* and *A. phagocytophilum* [12] despite the occurrence of co-infection in vertebrates. The detection of both pathogens confirmed co-infection in these animals with both *B. divergens* and *A. phagocytophilum*, the causative agent of tick-borne fever. Both these organisms share the same tick vector *I. ricinus*; however unlike babesiosis, clinical signs of tick-borne fever in cattle are usually mild and transient. In a study carried out in Swedish cattle, co-infection with both organisms was reported in almost 20% of the clinical cases [13]. Although the full clinical and epidemiological effects of these co-infections are poorly understood, it is thought they may exacerbate clinical illness and therefore may have been a contributing factor to the relatively high morbidity and mortality rate in this case.

**Fig. 2:**
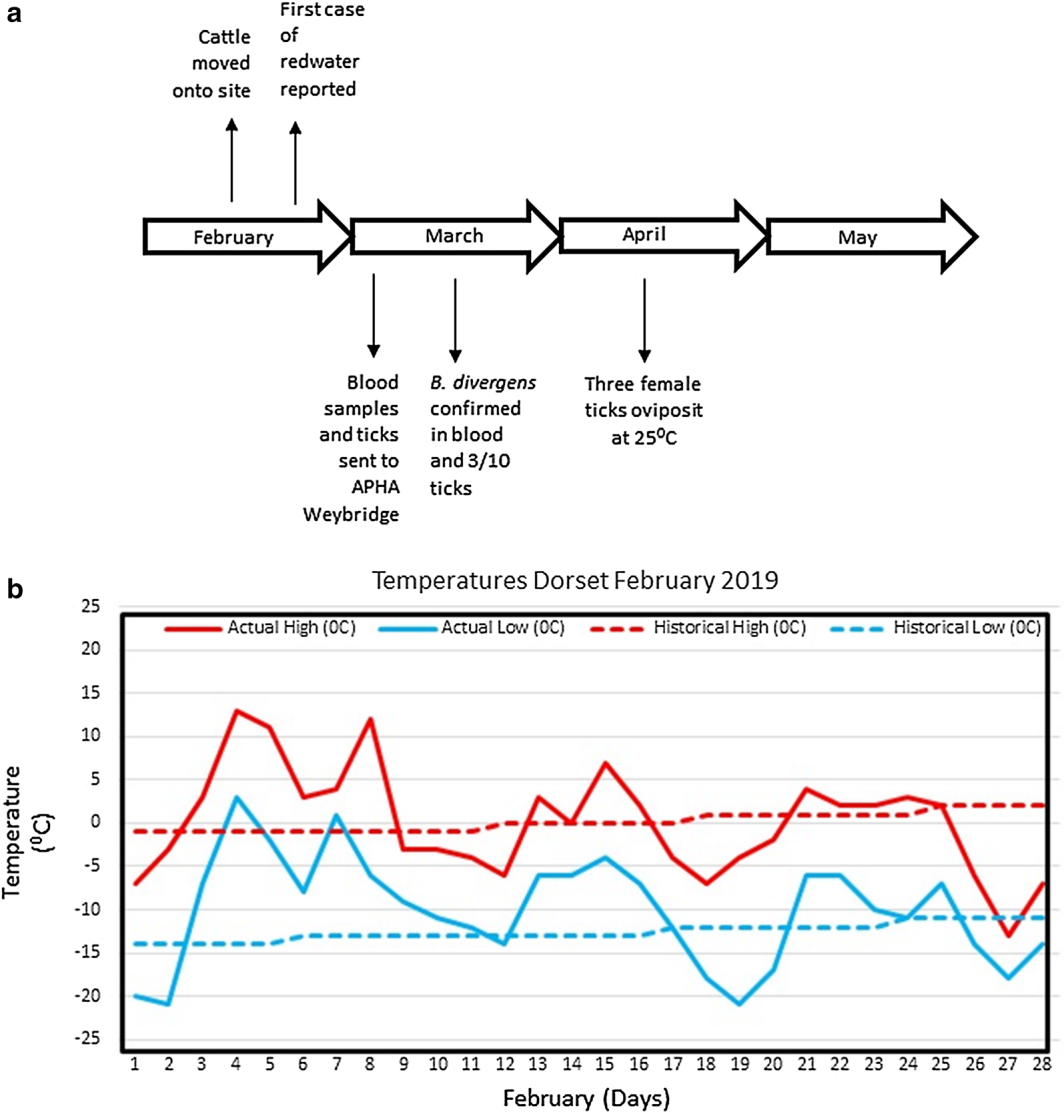
Timeline of bovine babesiosis outbreak in Dorset, UK. **a** A schematic timeline from the initial movement of cattle onto the tick-infested fields to the oviposition of some of the engorged ticks removed from the cattle. **b** The average and actual temperatures in Dorset in February

Cases of babesiosis in cattle in the UK usually occur between March and November, associated with the months when the tick vector is active, so an outbreak in February is considered early (Fig. 2a). To investigate this further, February temperature data for the county of Dorset was obtained [14] to assess whether there could be a link to the outbreak. This shows that based on historical averages, the maximum temperature is around 0 °C and the minimum lower at below − 10 °C. These temperatures would prevent tick activity and questing for a vertebrate host. However, in February, both the maximum and minimum temperatures were considerably higher than the average with a number of periods when the minimum temperature remained above 0 °C and with peak temperatures above 10 °C (Fig. 2b). It is possible that at these elevated temperatures, ticks actively started host-seeking behaviour leading to the tick-borne pathogen transmission. Observations of tick behaviour in Switzerland have demonstrated that a continuous period of five days with temperatures over 7 °C stimulated *I. ricinus* questing activity [15].

## Conclusions

It is possible that above average temperatures for southern England during what is considered a winter month may have triggered *I. ricinus* ticks to quest for a vertebrate host earlier than normal providing the opportunity for transmission of *B. divergens* and *A. phagocytophilum*. This combined with the movement of cattle onto the site, resulted in an early outbreak of babesiosis in this herd in February. This study also confirms the utility of using molecular methods, PCR and sequencing, to confirm the presence of a piroplasm infection and identify it to species. This has previously been shown for an outbreak of babesiosis in cattle [7], babesiosis in dogs [10] and detected *B. vogeli* in an imported dog [16]. The approach has also successfully detected *Theileria* spp. in the UK in sheep [17] and cattle [18]. Co-infection with *A. phagocytophilum* and tick-borne pathogens such as *B. divergens* and louping ill virus [19] has been shown to increase the severity of disease in livestock and should be considered when diagnosing clinical cases.

## Data Availability

The data supporting the conclusions are included in the article. The newly generated sequence was submitted to GenBank under Accession Number MT550684.
